# Editorial: Intensive management of cardiovascular disease patients with confirmed or suspected COVID-19 in area where Chagas disease is endemic

**DOI:** 10.3389/fmed.2023.1167877

**Published:** 2023-03-03

**Authors:** Marcelo Arruda Nakazone, Victor Sarli Issa, Reinaldo Bulgarelli Bestetti

**Affiliations:** ^1^Department of Cardiology and Cardiovascular Surgery, Faculdade de Medicina de São José do Rio Preto, São José do Rio Preto, Brazil; ^2^Department of Cardiology, Antwerp University Hospital, Antwerp, Belgium; ^3^Department of Medicine, Medical School, University of Ribeirão Preto, Ribeirão Preto, Brazil

**Keywords:** COVID-19, Chagas disease, cardiovascular disease, acute coronary syndrome (ACS), intensive management

In the current era, noncommunicable chronic diseases (NCD) continue to lead global statistics regarding causes of death, disabilities, and loss of quality of life, and culminate in great social and economic impacts worldwide. In Brazil, among the deaths resulting from NCD, nearly 30% are due to cardiovascular diseases (CVD) (1) as leading causes, as estimated by the last update from GBD observational study (https://www.healthdata.org/gbd/2019).

Cardiovascular involvement has been highlighted as a major risk factor for complications of coronavirus disease 2019 (COVID-19) since the beginning of the pandemic (2). Although the GBD 2019 study has also shown that the number of deaths from Chagas disease (ChD) in Brazil has decreased over time (from 7,903 in 1990 to 6,523 in 2019), when specifically analyzing the mortality of patients due to Chagas heart failure, this rate still represents around 7.8% of deaths (3).

Thus, considering that the spread of COVID-19 in ChD-endemic areas could have been a challenging issue in the context of public health policy, particularly due to higher risk for heart complications in co-infected patients, we aimed to address the burden of this condition during the worst waves of the pandemic. In fact, some important findings from different Brazilian regions have been captured in this Research Topic entitled “*Intensive management of cardiovascular disease patients with confirmed or suspected COVID-19 in area where Chagas disease is endemic*,” which has been successfully launched in Frontiers in Medicine.

First, Scolari et al. highlighted his team's experience in managing a cohort of 21 heart transplant recipients infected by COVID-19, of which 62% required hospitalization, between March 2020 and October 2021. Most of patients presented typical symptoms, there were no cases of immunosuppression withdrawal or acute graft rejection during the symptomatic period or in a short-term of follow-up. It is important to mention that only one of them had ChD concomitant to ischemic cardiomyopathy, with no post-transplant reactivation. The few patients already vaccinated and infected by COVID-19 survived and in-hospital mortality rate was comparable to other similar cohorts worldwide.

Following articles published in our Research Topic, Sperandio da Silva et al. presented the clinical course of 81 hospitalized patients due to COVID-19 complications—all with confirmed tests for ChD and ~25% of them successively readmitted—in the northeast region of Brazil. Regarding the total number of hospitalizations, the multiple logistic regression analysis suggested that ChD individuals hospitalized due to COVID-19 had around six-fold higher risk for in-hospital mortality compared to patients admitted due to other causes. Thus, although we must recognize its observational condition and the mentioned sample limitation, this is the largest study in this regard published so far. Furthermore, this investigation expands the previous view of another observational study, which showed an increase in the prevalence of acute heart failure and atrial fibrillation, but no effect on prognosis, in patients with COVID-19 and ChD (4).

After two original studies, Bestetti et al. proposed an extended literature review about the potential burden on morbidity and mortality of patients with coinfection—ChD and severe acute respiratory syndrome coronavirus 2. In this manuscript, the authors highlighted cardiovascular complications commonly observed in ChD patients (such as coronary artery and cerebrovascular diseases, heart failure, cardiac thrombosis and pulmonary embolism, cardiac arrhythmia, and sudden cardiac death) and provided some suggestions for the management of CVD in patients with COVID-19 in areas where ChD is endemic, where both conditions may further aggravate morbidity, mortality, and disability-adjusted life years of patients with cardiovascular involvements.

Finally, a last study accepted in our Research Topic aimed to evaluate the burden of COVID-19 on outcomes among patients prospectively admitted due to acute coronary syndromes (ACS) in three cardiology centers from Brasilia—an endemic region for Chagas and other infectious diseases—from May 2020 to February 2021. In this study, even recognizing the reduced number of ischemic events at the peak of the pandemic, the authors observed that COVID-19 predicted a higher risk for mortality among patients admitted with ACS, suggesting the severity of the two diseases when they occur concomitantly (Junior et al.).

In summary, these contributions shed further light into the potential cardiovascular complications in patients infected by COVID-19. We hope the reader of this Research Topic will be encouraged to appropriately manage not only patients with coronary artery disease, but especially those with ChD, an endemic but still neglected disease in Latin America, and now globalized because international immigration.

## Author contributions

MN conceived the idea. VI and RB critically reviewed the manuscript. All the authors have approved the manuscript in its final format.
